# Safety of Stereotactic Body Radiation Therapy for Seven Ipsilateral Lung Lesions

**DOI:** 10.7759/cureus.8759

**Published:** 2020-06-22

**Authors:** Mark E Bernard, Lana Critchfield, Mahesh Kudrimoti

**Affiliations:** 1 Radiation Oncology, University of Kentucky, Lexington, USA

**Keywords:** sbrt, multiple, lung

## Abstract

Our patient is a 48-year-old gentleman who initially was diagnosed with stage IV colorectal cancer on presentation. He had a history of multiple lines of systemic therapy, primary local resection, radioembolization treatments to the liver, along with liver metastasectomy with intraoperative radiofrequency ablation. He initially presented and completed single-isocenter stereotactic body radiation therapy (SBRT) to five right lung metastases with a treatment response and then completed SBRT to two additional right lower lung metastases. Since completion of his SBRT, he has remained disease free. Integration of SBRT to multiple ipsilateral lung lesions achieved a complete clinical response and assisted in keeping him disease free with the support of systemic therapy, and thus improved quality of life.

## Introduction

Growing evidence has shown radiation treatment to oligometastatic sites can improve progression-free survival (PFS) and suggests an improvement in overall survival (OS) [1]. Stereotactic body radiation therapy (SBRT) is a great local treatment option for oligometastatic cancer. It provides an ablative dose of radiation in one to five treatments and can be sequenced in-between chemotherapy, immunotherapy, or targeted therapy cycles. A recent National Surgical Adjuvant Breast and Bowel Project, Radiation Therapy Oncology Group, Gynecologic Oncology Group (NRG) study began in hopes of determining the safety and efficacy of SBRT treatment to four or less metastatic lesions [2]. Results from the study published by Chmura et al. demonstrated the safety of treating between two and four metastases with SBRT [3]. However, there is no consensus on the safety of treating greater than or equal to five lesions, the number of treatments that can be delivered, the safety of treating neighboring lesions, or of the delivery method of the SBRT treatments. We now present a case of a gentlemen who had five right lung lesions treated synchronously followed by two additional right lung lesions treated synchronously, to a total of seven lesions treated with SBRT in the same lung, less than 5 cm apart from each other.

## Case presentation

**Figure 1:**
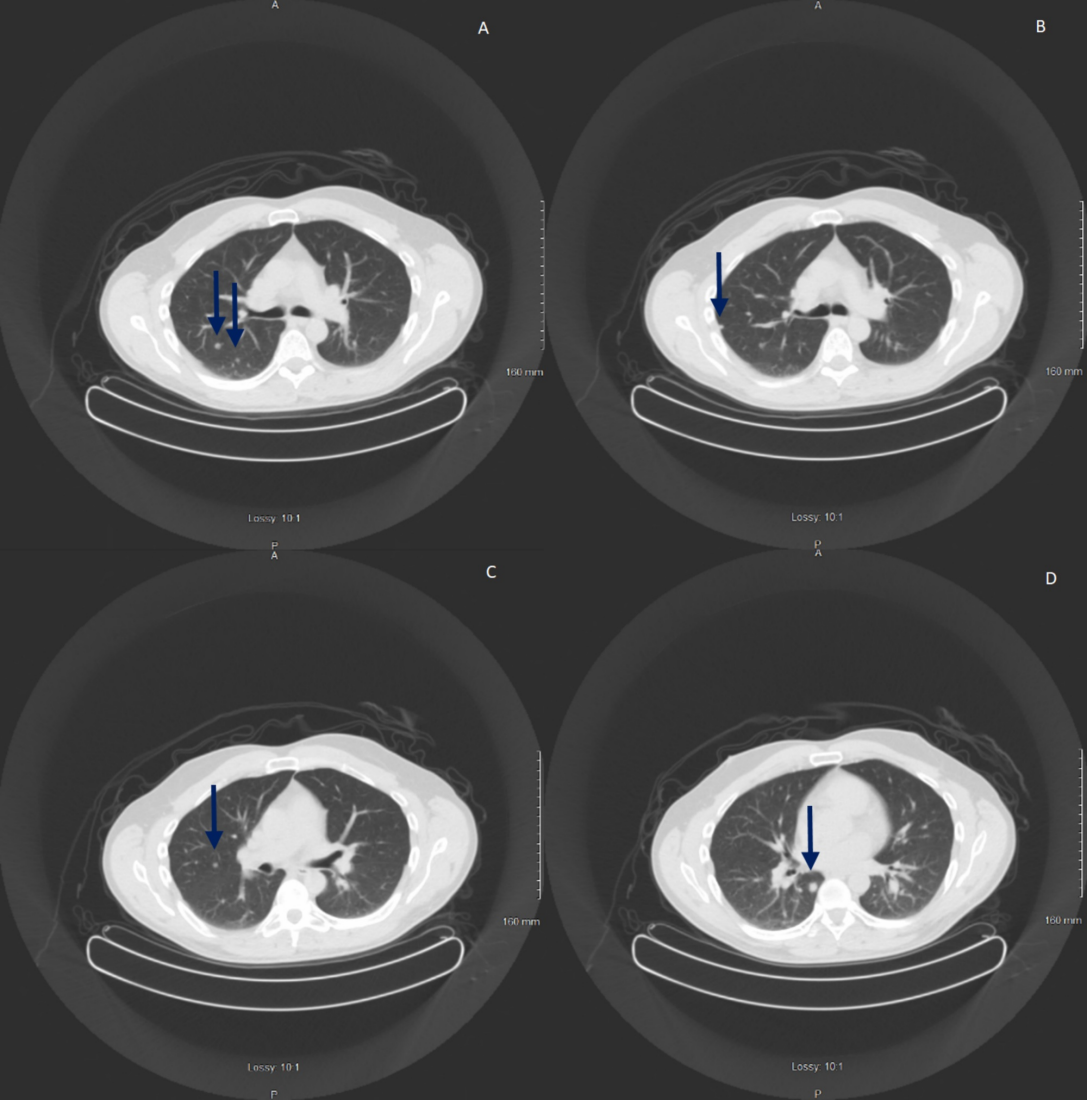
The figure shows the five lesions seen on the diagnostic CT scan consistent for metastatic colorectal cancer. A-D show the CT scan images and various locations.

Our patient was initially diagnosed with stage IV colon cancer with liver metastasis in 2015 and received folinic acid, fluorouracil, oxaliplatin, irinotecan (FOLFOXIRI) and had an emergent surgery secondary to bowel perforation with a sigmoid resection and was found to have a pT3N1 colon cancer. Afterwards he received FOLFOXIRI with bevacizumab with a good clinical response, and subsequent liver resection showed no evidence of malignancy. In 2016, he was found to have recurrent liver metastases and received a metastectomy of four lesions and intraoperative radiofrequency ablation (RFA). Later in 2016, he went to receive radioembolization to the right and left liver. In 2017, he was found to have multiple lung lesions and liver metastasis, and received RFA to a liver lesion, and was eventually placed on panitumumab. However, in 2018 he presented to Radiation Oncology at the University of Kentucky with multiple right-sided lung nodules for consideration of SBRT for his right lung nodules. Figure 1 shows the lung nodules on a diagnostic CT scan.

**Figure 2:**
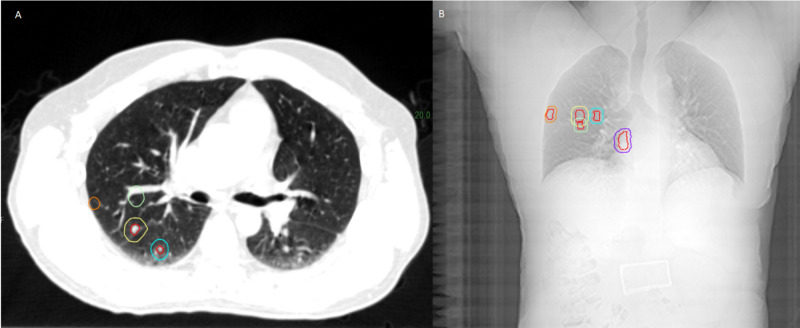
The figure shows the simulation CT scan and the digitally reconstructed radiographs of all the treated lesions. (A) Shows the lesions on the simulation CT scan. (B) Shows the lesions on the digitally reconstructed radiographs.

At the time of his CT simulation, he was placed in a Vac-Lok^TM^ (CIVCO, Orange City, IA) bag with wingboard, on a CIVCO SBRT board. A free-breathing scan was obtained with a Lightspeed 16-slice CT scanner (General Electric Medical Systems, Waukesha, WI) with 512 x 512-pixel image size and 2.5-mm slice thickness in the axial helical mode. A four-dimensional CT scan utilizing the Varian Real-time Position Management (RPM; Varian Medical Systems, Palo Alto, CA) system was used to create a maximum intensity projection (MIP) scan. In the Eclipse treatment planning system (Varian Medical Systems, Palo Alto, CA), the MIP scan was fused to the free-breathing CT scan. The gross tumor volume (GTV) was determined from the free-breathing CT scan, and the internal target volume (ITV) was determined by the MIP from the four-dimensional CT scan. An isotropic margin of approximately 4 mm was added to create the planning target volume (PTV). A total of five GTVs/ITVs were created with corresponding PTVs. The volumes were as follows: PTV1 = 2.98 cm^3^, PTV2 = 7.75 cm^3^, PTV3 = 3.98 cm^3^, PTV4 = 4.24 cm^3^, and PTV5 = 9.74 cm^3^. Figure 2 shows the lesions to be treated.

**Figure 3:**
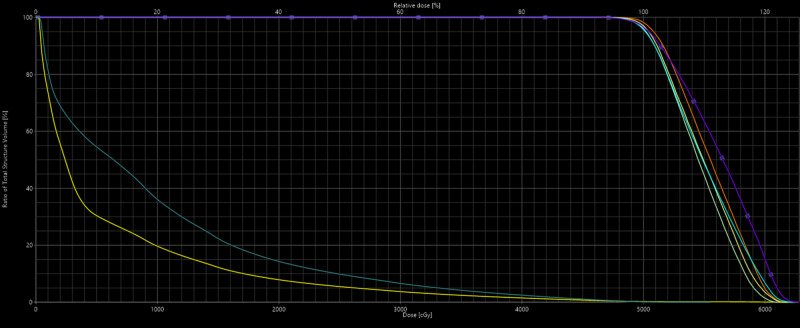
The figure shows the dose-volume histogram for the first SBRT plan. The yellow line is the bilateral lung-PTV and the darker green line is the right lung-PTV. SBRT, stereotactic body radiation therapy; PTV, planning target volume.

All five lesions received 50 Gy in five fractions. PTVs were planned to the 80% isodose line and optimized such that 95% of each PTV received 100% of the prescription dose, resulting in a hotspot of approximately 120% of prescription dose in the center of each GTV. Treatment planning consisted of a single-isocenter placed approximately between the five lesions. Volumetric-modulated arc therapy (VMAT) was planned with three partial arcs from 180° to 30° with 6 MV flattening filter free (6X-FFF). Collimator angles were chosen to reduce the multileaf collimator (MLC) interleaf leakage in order to reduce normal lung dose. Daily cone-beam CT scans were utilized for each treatment. On the dose-volume histogram (DVH), the right ipsilateral lung V20-PTV was 14%, but the bilateral lung-PTV V20 was 8%. For all five of PTVs, D95 = 100%. The DVH is seen in Figure 3.

**Figure 4:**
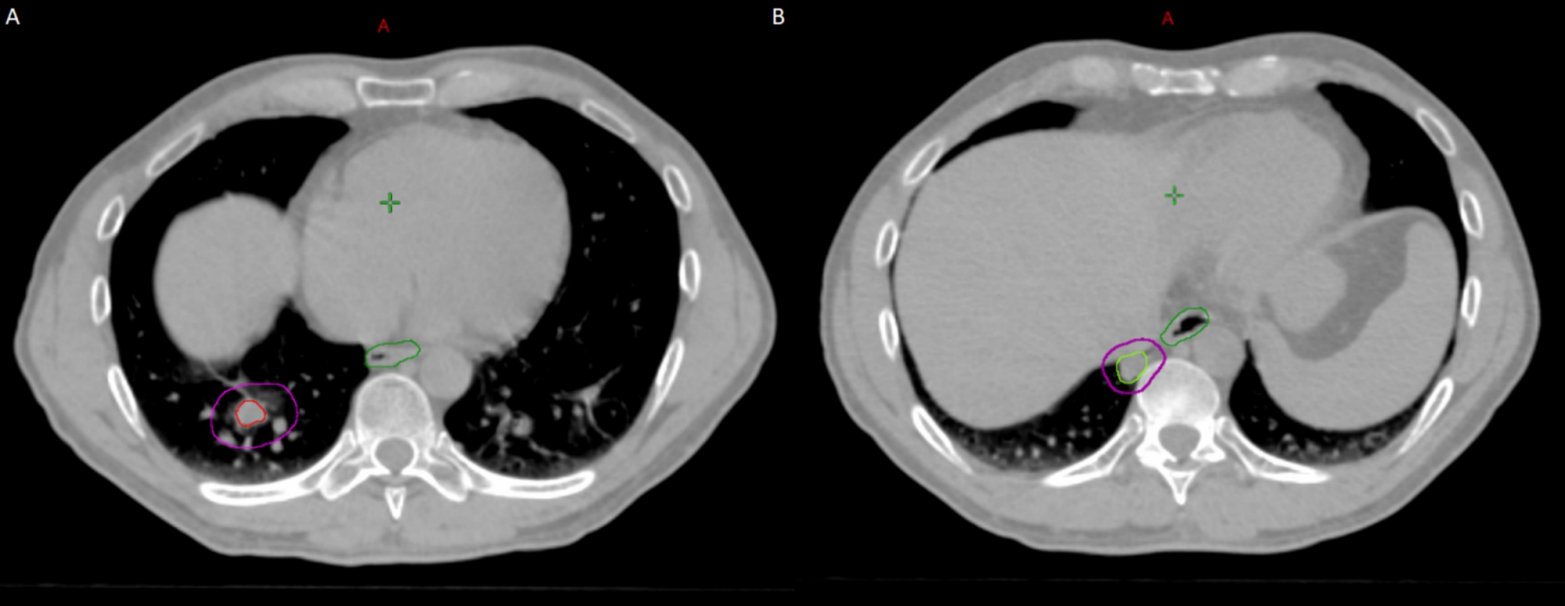
The planning CT scan for the second course of SBRT. They were treated with a single isocenter technique. (A) PTV1 was treated with 50 Gy in five fractions. (B) PTV2 was treated with 45 Gy in five fractions given the close proximity to the spinal cord and esophagus. SBRT, stereotactic body radiation therapy; PTV, planning target volume.

His chest CT scan done eight weeks later showed a decrease in the right lung nodules. However, he had new progression of two right lower lung nodules in the right lower lobe. A positron emission tomography/CT (PET/CT) scan confirmed the presence of these right lung metastasis and he was referred back to Radiation Oncology at the University of Kentucky for further management. We then simulated and planned the patient in a similar fashion as before and treated the two right lower lobe lesions synchronously using 50 Gy in five fractions (PTV1 = 22.57 cm^3^) and 45 Gy in five fractions (PTV2 = 13.28 cm^3^). A combined DVH was created, which included the prior SBRT plan, and showed the bilateral lung-PTV was 10% and the right lung-PTV was 21%. Figure 4 shows the planning CT for his second SBRT treatment.

**Figure 5:**
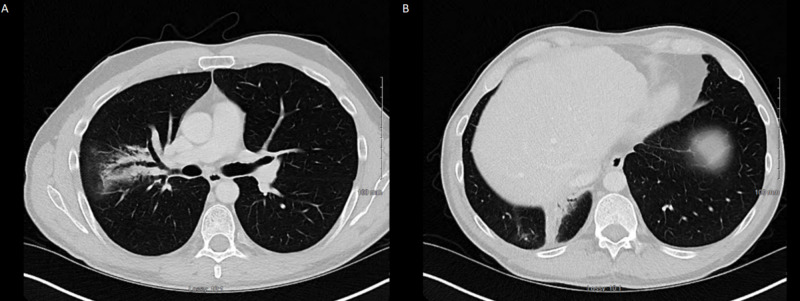
The figure shows the chest CT scan after a 14-month interval from his last SBRT treatment. It shows post-radiation changes and no evidence of progression. He also had no other sites of metastatic disease. (A) Shows radiation changes in the upper right lung. (B) Shows radiation changes in the lower right lung. SBRT, stereotactic body radiation therapy.

The post-treatment scan done one month after showed a decrease in the right lower lung nodules, and subsequent CT scans showed resolution of all pulmonary nodules with post-radiation changes. He was eventually placed back on systemic therapy 11 days later. His most recent restaging chest, abdomen, and pelvic CT scans done 14 months later showed no evidence of metastatic disease. He also had no respiratory symptoms. Figure 5 shows his most recent chest CT scan.

## Discussion

SBRT has been shown to be an efficacious treatment for patients with oligometastatic cancer in both retrospective and prospective reports [1,4]. In 2019, the Stereotactic Ablative Radiotherapy for the Comprehensive Treatment of Oligometastatic Disease (SABR-COMET) trial was the first known prospective trial showing a clinical benefit of SBRT in the oligometastatic setting [1]. Patients in this trial had a controlled primary tumor with up to five metastatic lesions. The control arm received palliative standard of care and the experimental arm had the addition of SBRT. The addition of SBRT resulted in a median PFS benefit from 6 to 12 months and was associated with an OS benefit from 28 months to 41 months. Unfortunately, there was a 4.5% grade toxicity rate. While this trial allowed up to five lesions, the majority had one to two lesions, and there is currently no published prospective trial allowing more. However, the daughter trial is expected to allow four to ten lesions [5]. Our patient clearly has had a long progression-free interval, with the use of SBRT, which correlates with the results of this trial.

NRG-BR001 is a prospective clinical trial also allowing the use of SBRT in the oligometastatic setting up to four lesions [2]. There are a variety of fraction schedules allowed based upon the location. However, the peripheral lung lesions are given 45 Gy in three fractions and the central lung lesions are given 50 Gy in five fractions. This trial allowed for lesions to be treated within 5 cm of each other, while recognizing that toxicity and safety of SBRT is limited for lesions within 5 cm of each other. For this patient, four out of the five lesions were within 3 cm of each other during the first treatment. Both lesions were within 3 cm of each other during the second treatment. Despite the close proximity of all these lesions, our patient did not experience concerning side effects. However, our PTVs were not large and thus the amount of treated lung is small and could also play a factor in his performance status.

In 2014, Li et al. published their experience treating one patient with five lung lesions and one patient with seven lung lesions with 48 Gy in eight fractions (biologically effective dose [BED] 77 Gy) and 42 Gy in seven fractions (BED 67 Gy), respectively [6]. These lung lesions were bilateral. Both patients did well with grade 1 pneumonitis in the latter patient; however, both patients had a slight deterioration in pulmonary function at a six-month follow-up. Our case report differs from this report in that all lesions were within the same lung and we have 14-month follow-up showing no progression and no concerning late side effects. However, similar to this case report, our patient did not develop concerning respiratory side effects.

Wegner et al. published a series of 22 patients who received lung SBRT for colorectal metastasis [7]. The median dose was 48 Gy in five fractions. There were no grade 3 side effects and the one-year and the three-year local control rates were 75% and 58%, respectively. However, the median number of metastasis was 1 (range, 1-3). Our experience is unique from this study in that we treated five lesions in a single session followed by two lesions shortly afterwards. However, this study and our case report show that SBRT is a feasible option for patients with lung metastasis from a colorectal primary and can achieve a long-term disease-free interval.

This case report is unique in many respects. All lesions were present in the right lung and were treated synchronously utilizing single-isocenter VMAT SBRT, and we have a 14-month follow-up showing the patient is completely disease free. We were able to keep the total lung V20 below 10% and there was only grade 1 (radiographic) pneumonitis at that last follow-up. We also showed lesion proximity did not affect our patient. Due to this, we propose the idea that dose constraints being met to the organs at risk may override the concern for proximity and number of synchronously treated lesions. However, we recognize that our PTVs were not large and should be taken into consideration. The small size of the PTVs could be the primary reason why the side effect profile was low and it is recognized to be a limitation of our case report conclusions.

## Conclusions

This case report shows SBRT in the oligometstatic setting can be safely given for seven neighboring lesions in a short period of time, and can assist in providing a disease-free interval with systemic therapy, with a good safety profile. Therefore, SBRT should and can be used more in the metastatic setting, especially for patients attempting to achieve a prolonged disease-free survival interval.
